# Integrating environmental DNA monitoring to inform eel (*Anguilla anguilla*) status in freshwaters at their easternmost range—A case study in Cyprus

**DOI:** 10.1002/ece3.9800

**Published:** 2023-02-27

**Authors:** Nathan P. Griffiths, Rosalind M. Wright, Bernd Hänfling, Jonathan D. Bolland, Katerina Drakou, Graham S. Sellers, Stamatis Zogaris, Iakovos Tziortzis, Gerald Dörflinger, Marlen I. Vasquez

**Affiliations:** ^1^ Biological and Marine Sciences, Hardy Building University of Hull Hull UK; ^2^ Environment Agency Feering UK; ^3^ Institute for Biodiversity and Freshwater Conservation University of the Highlands and Islands Inverness UK; ^4^ Department of Chemical Engineering Cyprus University of Technology Limassol Cyprus; ^5^ Hellenic Centre for Marine Research Institute of Marine Biological Resources and Inland Waters Anavissos Greece; ^6^ Water Development Department, Ministry of Agriculture Rural Development and Environment Nicosia Cyprus

**Keywords:** catadromous, connectivity, dams, distribution, eDNA, eel management plans, intermittent, island, Mediterranean, policy

## Abstract

Despite significant population declines and targeted European Union regulations aimed at *Anguilla anguilla* conservation, little attention has been given to their status at their easternmost range. This study applies wide‐scale integrated monitoring to uncover the present‐day eel distribution in Cyprus' inland freshwaters. These are subject to increasing pressures from water supply requirements and dam construction, as seen throughout the Mediterranean. We applied environmental DNA metabarcoding of water samples to determine *A. anguilla* distribution in key freshwater catchments. In addition, we present this alongside 10 years of electrofishing/netting data. Refuge traps were also deployed to establish the timing of glass eel recruitment. These outputs are used together, alongside knowledge of the overall fish community and barriers to connectivity, to provide eel conservation and policy insights. This study confirm the presence of *A. anguilla* in Cyprus' inland freshwaters, with recruitment occurring in March. Eel distribution is restricted to lower elevation areas, and is negatively associated with distance from coast and barriers to connectivity. Many barriers to connectivity are identified, though eels were detected in two reservoirs upstream of dams. The overall fish community varies between freshwater habitat types. Eels are much more widespread in Cyprus than previously thought, yet mostly restricted to lowland intermittent systems. These findings make a case to reconsider the requirement for eel management plans. Environmental DNA‐based data collected in 2020 indicate that “present‐day” eel distribution is representative of 10‐year survey trends. Suggesting that inland freshwaters may act as an unrealized refuge at *A. anguilla's* easternmost range. Conservation efforts in Mediterranean freshwaters should focus on improving connectivity, therefore enabling eels to access inland perennial refugia. Thus, mitigating the impact of climate change and the growing number of fragmented artificially intermittent river systems.

## Graphical abstract

This study applies wide‐scale eDNA and catch‐based monitoring to confirm present‐day eel status in Cyprus' inland freshwaters. These methods drew parallel conclusions, finding that eel distribution is mostly restricted to lowland coastal areas, and is negatively associated with migration barriers on the island.

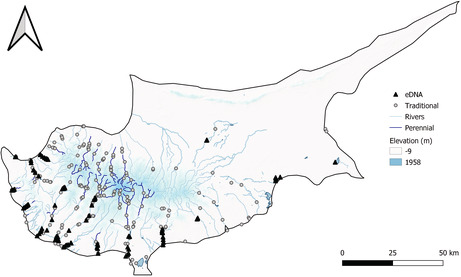

## 1 INTRODUCTION

The European eel (*Anguilla anguilla*) is a catadromous fish species which spawns in the Sargasso Sea (Schmidt, 1923). Over the last four decades, significant global declines in eel populations have been observed (Aalto et al., 2016; Bilotta et al., 2011; Correia et al., 2018; Jacoby & Gollock, 2014; Podgorniak et al., 2016). *Anguilla anguilla* recruitment is now estimated to be <10% of what was recorded in the 1970s (ICES, 2019; Trancart et al., 2020), and faces a myriad of pressures including migration barriers, habitat loss, turbine/pump mortality, overfishing/illegal exploitation, and climate change (Acou et al., 2008; Bilotta et al., 2011; Bolland et al., 2019; Buysse et al., 2015; Dekker, 2000; Moriarty & Dekker, 1997; Økland et al., 2019; Trancart et al., 2020). In response, the IUCN has classified *A. anguilla* as ‘critically endangered’ (Jacoby & Gollock, 2014), and the European Union has implemented specific legislation [The EC Eel Regulation (1100/2007)], requiring member states to develop eel management plans (EMPs). These regulations aim to facilitate the recovery of *A. anguilla* by implementing EMPs with the aim to achieve >40% of historic silver eel biomass (prior to anthropogenic impacts) safe passage (escapement) on their spawning migration between inland waters and the sea (Aalto et al., 2016; Council of the European Union, 2007). *A. anguilla* is distributed throughout the Mediterranean, and this region could provide a significant contribution to overall species recovery (Aalto et al., 2016). More recently, the General Fisheries Commission for the Mediterranean (GFCM) adopted the Recommendation GFCM/42/2018/1 (GFCM, 2018), establishing more targeted management measures for *A. anguilla* in the Mediterranean Sea (ICES, 2021). However, because the systematic investigation of eels in Cyprus' freshwaters only commenced approximately a decade ago (Zogaris et al., 2012), the present‐day eel status and distribution on the island is subject to uncertainty.

Intermittent rivers and ephemeral streams (IRES), defined as watercourses which cease to flow at some point in time and space (Datry et al., 2018), are among the most common freshwater ecosystems globally (Larned et al., 2010). Furthermore, due to drying climates and increased human pressures on freshwaters, these systems are increasing in number, particularly in semi‐arid regions (Datry et al., 2014, 2018). This has been noted in Mediterranean lotic systems, where increasing climate and anthropogenic pressures are leading to artificially intermittent river systems (Skoulikidis et al., 2011). The partial or complete drying of these systems is detrimental to whole fish communities, however upon flow resumption, fish recolonization from upstream perennial reaches has been observed (Skoulikidis et al., 2011). The nature of these systems poses challenges for the implementation of eel management policy, since migration trigger flows may be present, but with no guarantee of perennial freshwater refuge (Zogaris et al., 2012).

Our case study area, the Mediterranean island of Cyprus, is part of the eastern limit of *A. anguilla*'s range. Until recently (2011 onwards), quantitative eel catch data from the island's freshwaters were lacking (Zogaris et al., 2012). Therefore, the island was exempted from any obligation to implement eel management plans in 2009 (2009/310/EC). Through the Water Framework Directive (WFD) fish monitoring program, however, more recent data have suggested that *A. anguilla* may be more widespread in Cyprus than previously thought (Zogaris et al., 2012). Here, Zogaris et al. (2012) found that eels were the most widespread native fish species remaining in the island's freshwaters when conducting site‐specific electrofishing, expert interviews, and literature reviews. Yet, when omitting data obtained from expert interviews and literature reviews, the observed *A. anguilla* occurrence was very low and localized. Cyprus represents a typical Mediterranean island, where lotic freshwater systems are often dominated by intermittent rivers and ephemeral streams (Papastergiadou et al., 2016). The historic natural state of most freshwater systems is not known, as anthropogenic impacts, namely water diversion and groundwater abstraction, have hugely impacted the freshwaters of the semi‐arid island in recent years (Markogianni et al., 2014). Indeed, the inland freshwaters are influenced by an estimated 108 dams, one of the highest densities of dam reservoirs in Europe (Zogaris et al., 2012). Changes in flow regimes may force eels into summer refugia earlier, but with water retained upstream of likely impassible retention dams there is risk of restricting perennial habitat, while inducing artificial intermittence downstream (Skoulikidis et al., 2011). Despite these interruptions to natural flow regimes, there are catchments with perennial streams, particularly on the more humid western side of the island, and at higher elevations (Dörflinger, 2016; Papastergiadou et al., 2016; Zogaris et al., 2012).

While lentic inland freshwater habitat is present in Cyprus, natural wetlands are particularly vulnerable to climate change and anthropogenic impacts, yet artificial freshwaters are not generally managed with conservation values as a priority (Markogianni et al., 2014). A study by Markogianni et al. (2014) found that most natural freshwater wetlands are smaller than artificial sites, and ephemeral in nature, with only eight of the 30 largest wetlands classified as naturally occurring. Relating back to artificially intermittent streams (Skoulikidis et al., 2011), the presence of perennial wetlands has the potential to act as summer refuge habitat for fish species. This, of course, requires an element of—at least temporary—connectivity, and an assumption that wetland habitats are maintained year‐round. Historically, such habitats were not valued as they were viewed as impediments to development and potential host areas for disease (Markogianni et al., 2014). The current situation in Cyprus and other Mediterranean countries, is that natural wetlands are threatened by development and water use pressures (e.g., abstraction for irrigation), while artificial sites are not always managed with conservation values in mind.

In order to understand the value of Mediterranean freshwaters for eels, it is essential that we first have an improved understanding of the present‐day eel status and distribution. To assess the eel distribution in Cyprus, and therefore inform current status, a wide range of catchments must be monitored in a short time frame. Environmental DNA (eDNA) metabarcoding of water samples has already proven to be effective for monitoring *A. anguilla* in heavily modified river systems in the UK (Griffiths et al., 2020). Therefore, this case study builds upon current knowledge by applying eDNA metabarcoding of water samples taken in 2020, alongside data from electrofishing/netting surveys carried out over a 10‐year period (2009–2019) as part of the national WFD monitoring program. By applying integrated monitoring methods, novel information on recent trends, and a snapshot of present‐day fish distribution can be used to facilitate the improved understanding of *A. anguilla* distribution drivers. With multiple monitoring methods to inform this in such heavily managed and variable catchments, we aim to: (a). Determine the current eel status and distribution in Cyprus; (b). Assess the factors which influence eel distribution; and (c). Consider the wider implications for Mediterranean freshwaters.

## 2 METHODS

### 2.1 Environmental DNA metabarcoding (2020)

#### 2.1.1 Sample collection

**FIGURE 1:**
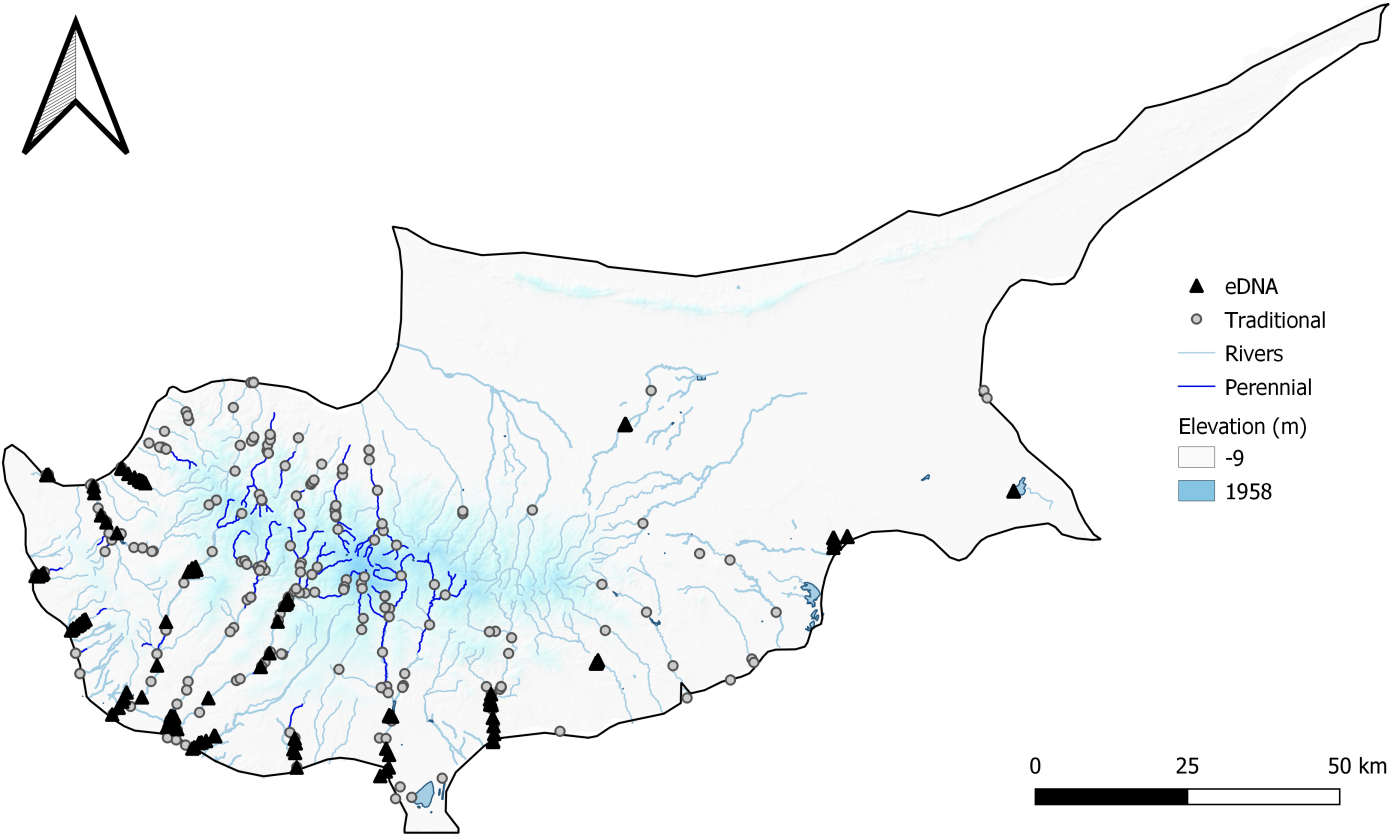
The distribution of data points across Cyprus, including the 130 eDNA sampling points in 2020 (black triangle) and the 299 fish survey points from 2009–2019 (grey dot). Areas of higher elevation are shaded in blue.

Twenty‐six inland freshwater sites were targeted for eDNA surveys, with 5 × (1.5 L) water samples taken from each between 04/02/2020 and 07/03/2020 (Figure 1). These sites were selected to include a representative range of freshwater habitat types, including; rivers (outlets) downstream of dams (*n* = 9), reservoirs upstream of dams (*n* = 9), unregulated rivers (*n* = 3), lentic wetlands (*n* = 3), and perennial spring‐fed sites (*n* = 2) (Table 1). Based on recent assessments, and excluding reservoirs where water is retained by dams, only two wetland sites, both spring‐fed sites, and one unregulated river were classified as perennial. The remaining systems have varying degrees of intermittence, although it is likely there are perennial refugia with unknown levels of connectivity in these catchments. Each of the five spatial replicate samples taken at each site consisted of 5 × 300 mL surface sub‐samples. A field/filtration blank was taken out into the field for each site, and processed alongside samples to monitor for contamination. Wherever possible, spatial replicates were taken at equidistant points spanning each study site (along the length of rivers, and perimeter of reservoirs/wetlands); however, where access did not allow this, we adhered to the following:

1. Rivers—Working from downstream to upstream, samples were taken at accessible points.
2. Reservoirs—When access was poor, sampling points were concentrated around the outlet where surface water was flowing suggesting increased mixing.

**TABLE 1:** An overview of the 26 sites where eDNA surveys were completed.

Site name	Code	Water body	Habitat type	Perennial (Y/N)	Avg water temp (°C)	Sampling date	Eel eDNA
Argaki tou Pyrgou (Aphrodite Baths)	AB	River	Spring Fed	Yes	18	11/02/20	Present
Argaka Dam (Makounta river)	AD	Reservoir	Reservoir	Yes	14.6	11/02/20	Present
Ovgos river (Avakas Gorge)	AG	River	River	Yes	14.8	18/02/20	Present
Asprokremmos Reservoir (Xeros river)	AP	Reservoir	Reservoir	Yes	15.3	04/02/20	Absent
Xeropotamos	AR	River	Outlet	No	13.1	11/02/20	Absent
Avdimou River	AV	River	River	No	15.3	16/02/20	Present
Verki coast (Agriokalami river)	BE	Coastal	Spring fed	Yes	14.2	17/02/20	Present
Cha‐Potami Reservoir (Cha‐Potami river)	CD	Reservoir	Reservoir	Yes	15.2	16/02/20	Present
Chrysochous River	CH	River	Outlet	No	12.8	11/02/20	Present
Cha‐Potami River	CP	River	Outlet	No	14.4	16/02/20	Present
Dhiarzos	DZ	River	Outlet	No	15	07/02/20	Present
Ezousa River	ES	River	Outlet	No	14.6	06/02/20	Present
Koshatis river (Ezousa river Tributary)	ET	River	River	No	17.3	05/02/20	Absent
Germasogeia river (Amathos)	GR	River	Outlet	No	13.3	18/02/20	Absent
Germasogeia Reservoir (Germoasogeia river)	GU	Reservoir	Reservoir	Yes	13.6	18/02/20	Absent
Kalavasos Reservoir (Vasilikos river)	KA	Reservoir	Reservoir	Yes	14	17/02/20	Absent
Arminou Reservoir (Diarizos river)	KD	Reservoir	Reservoir	Yes	11.3	12/02/20	Absent
Kannaviou Reservoir (Ezousa river)	KN	Reservoir	Reservoir	Yes	14.4	06/02/20	Absent
Kouris dam Outlet	KO	River	Outlet	No	14.1	17/02/20	Absent
Kouris Reservoir (Kouris river)	KU	Reservoir	Reservoir	Yes	14.4	17/02/20	Absent
Limni Mangli	MA	Lake	Wetland	Yes	N/A	16/02/20	Absent
Mavrokolympos Reservoir (Mavrokolympos river)	MD	Reservoir	Reservoir	Yes	14.3	13/02/20	Absent
Mavrokolympos Outlet	MR	River	Outlet	No	14.4	12/02/20	Absent
Oroklini Lake	OR	Lake	Wetland	Yes	14	17/02/20	Present
Paralimni lake	PA	Lake	Wetland	No	N/A	07/03/2020*	Absent
Xeros (Paphos district)	XE	River	Outlet	No	16	04/02/20	Absent

* Indicates sampling was outside of the refuge trap period.

Measures were put into place in the field to avoid contamination, samplers wore sterile gloves while handling sampling equipment, which were changed between samples. Sampling bottles were only opened for the first time when at site prior to sampling, and samples were transported in a bleach sterilized coolbox on ice, to be filtered within 24 h of collection. Following sample collection, water temperature was also recorded at each site (Table 1).

#### 2.1.2 Filtration

Upon returning to the Cyprus University of Technology (CUT) laboratory, 1 L of water from each sample was vacuum‐filtered through sterile 0.45 μm cellulose nitrate membrane filters with pads (47 mm diameter; Whatman, GE Healthcare, UK), using 2 filters per sample to reduce filter clogging. Prior to filtration all surfaces and equipment were sterilized with 10% bleach and 70% ethanol. Between filtration runs, filtration units were immersed in 10% bleach solution for 10 min, soaked in 5% v/v MicroSol detergent for an additional 10 min, and then rinsed thoroughly with purified water. Upon completion, filters were removed from units with sterile tweezers and placed back‐to‐back in 5 mL polypropylene screw‐cap tubes (Axygen, Fisher Scientific UK Ltd.), enclosed in sterile grip seal bags, and stored in a dedicated freezer at −20°C until DNA extraction. Filtration blanks (purified water) were processed alongside samples each day to monitor for contamination at this stage.

#### 2.1.3 DNA extraction

DNA from duplicate filters was co‐extracted following the Mu‐DNA: Water protocol (Sellers et al., 2018), with minor changes resulting from the existing laboratory set up (Appendix S1). As before, all surfaces were sterilized with 10% bleach and 70% ethanol, and all extraction reagents and plastics UV irradiated for 20 min prior to use. An extraction blank was included with each extraction run to monitor for contamination at this stage. To assess DNA quantity and purity, 2 μL aliquots of each DNA sample were analyzed on a Nanodrop 1000 Spectrophotometer (Thermo Fisher Scientific). Once completed, DNA extracts were stored at −20°C until PCR amplification.

#### 2.1.4 Library preparation

Library preparation was carried out following an established 12 S metabarcoding workflow previously developed at the University of Hull, the full protocol applied in this study can be viewed in Appendix S1, however, is summarized below:

Nested metabarcoding of DNA samples using a two‐step PCR protocol was performed at CUT, using multiplex identification (MID) tags in the first and second PCR steps as described in Kitson et al. (2019). PCR1 was performed in triplicate (3x PCR replicates per sample), amplifying a 106 bp fragment using published 12 S ribosomal RNA (rRNA) primers 12 S‐V5‐F (5′‐ACTGGGATTAGATACCCC‐3′) and 12 S‐V5‐R (5′‐TAGAACAGGCTCCTCTAG‐3′) (Kelly et al., 2014; Riaz et al., 2011). Alongside DNA extracts PCR‐negative controls (Molecular Grade Water) were used throughout, and positive controls (DNA (0.05 ng μL^−1^) from the non‐native cichlid *Maylandia zebra*) were added to each plate outside of the eDNA prep area. All PCR replicates from each plate were pooled together to create sub‐libraries and purified with MagBIND RxnPure Plus magnetic beads (Omega Bio‐tek Inc.), following a double size selection protocol (Quail et al., 2009). Following this, a second shuttle PCR (PCR2) was performed on the PCR1 cleaned products to bind uniquely indexed Illumina adapters to each sub‐library. A second purification was then carried out on the PCR2 products with the Mag‐BIND RxnPure Plus magnetic beads (Omega Bio‐tek Inc.). Eluted DNA was then stored at 4°C until quantification and normalization. After normalization and pooling, the final library was then purified again (following the same protocol as the second clean‐up), quantified by qPCR using the NEBNext Library Quant Kit for Illumina (New England Biolabs Inc., Ipswich, MA, USA) and diluted to 4 nM. The final library was then loaded (with 10% PhiX) and sequenced on an Illumina MiSeq using a MiSeq Reagent Kit v3 (600 cycle) (Illumina Inc.) at CUT.

Sub‐library sequence reads were demultiplexed to sample level using a custom Python script. Tapirs, a reproducible workflow for the analysis of DNA metabarcoding data (https://github.com/EvoHull/Tapirs), was then used for taxonomic assignment of demultiplexed sequence reads. Reads were quality trimmed, merged, and clustered before taxonomic assignment against a curated national fish reference database. Taxonomic assignment used a lowest common ancestor approach based on basic local alignment search tool (BLAST) matches with minimum identity set at 98%. The full bioinformatics workflow is detailed in Appendix S1.

### 2.2 Refuge traps

Alongside eDNA sampling, refuge traps were placed near the tidal limits downstream of potential barriers to upstream migration in key freshwater catchments (those with freshwater flows reaching the sea). These traps were made up from 2× domestic mop heads (Pop Life, Paphos), tied together and secured in‐stream. Refuge traps were checked regularly (every 2 to 4 days), lifted from the watercourse and emptied into a bucket to check for the presence of glass eels. These were deployed in key catchments and monitored from mid‐March to mid‐April 2019, and throughout January and February 2020.

### 2.3 National fish monitoring program (2009–2019)

These data (provided by Cyprus' Water Development Department) were primarily collected using in‐stream backpack electrofishing surveys to determine the fish species present. In cases where electrofishing was not possible, nets were used to enable fish assessment. The final dataset over this period includes 299 spatial and temporal surveys, largely focused in the more humid western region, but also covering more of the higher elevation central regions of the island (Figure 1). In addition to presence‐absence data, the measurements of captured fish were taken, providing size data for the 355 individual eels. Since these surveys were carried out with WFD assessments in mind, they are not grouped into freshwater habitat type in the way eDNA surveys were, and so for the purpose of this study are considered as individual survey points.

### 2.4 Data analysis and visualization

All downstream analyses were carried out using R version 3.6.3 (R Core Team, 2019). A low‐frequency read threshold of 0.001 was applied to eDNA data, removing any reads which make up <0.1% of total reads assigned to any given sample as previously applied in other studies using this 12 S marker (Griffiths et al., 2020; Handley et al., 2019; Hänfling et al., 2016). All maps were visualized, and associated metadata was extracted using QGIS (QGIS Development Team, 2022). Data were screened for normality using base R. Since our data did not conform to a normal distribution, they were subsequently tested for associations using Spearmans correlation tests (McDonald, 2014), and visualized using ggpubr (Kassambara, 2020). Correlation tests were carried out to determine associations between eel reads/catch and elevation, distance from coast, and number of migration barriers. All other plots were visualized using ggplot2 (Wickham, 2016).

## 3 RESULTS

### 3.1 eDNA metabarcoding

**FIGURE 2:**
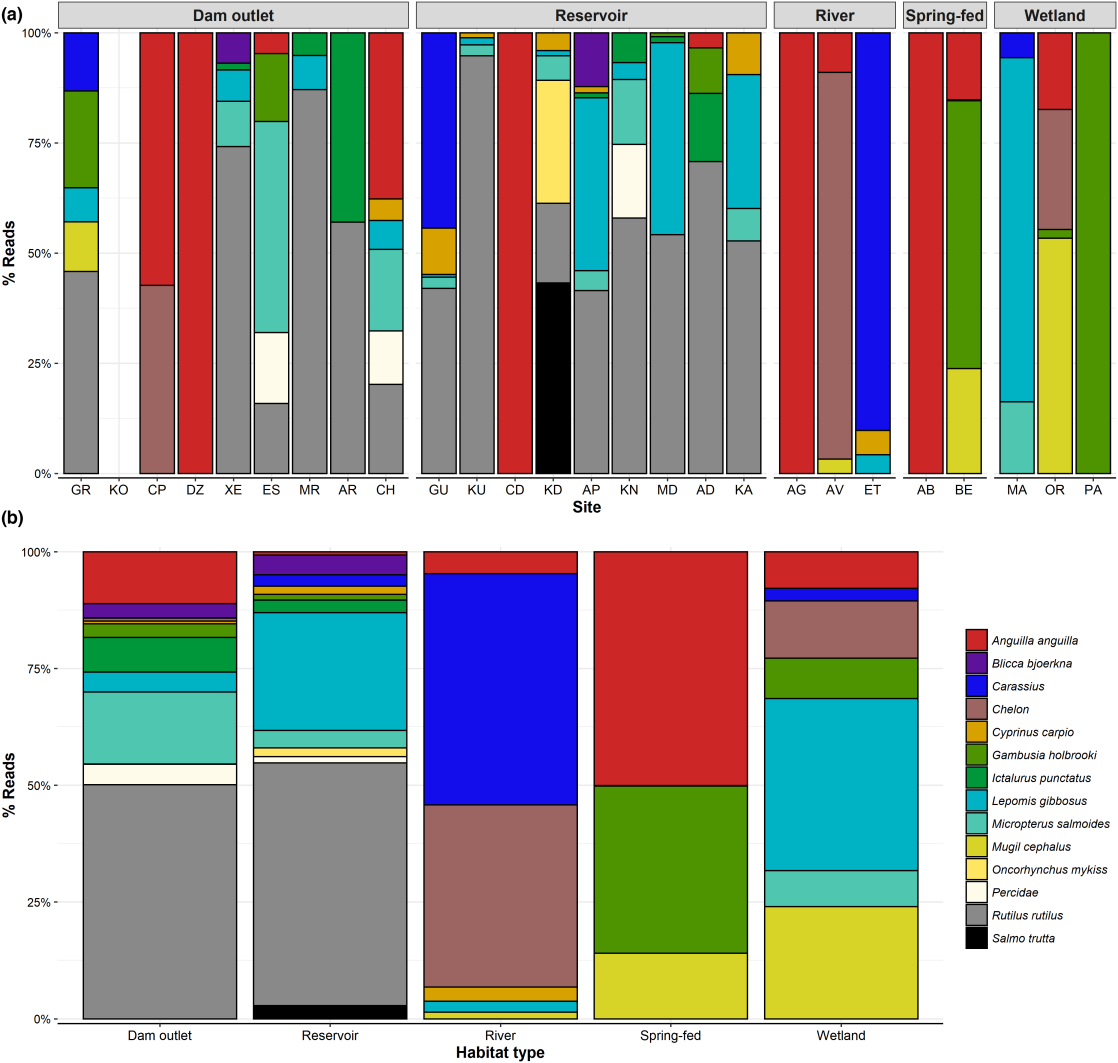
The relative abundance (% Reads) for each species detected, grouped into 5 freshwater habitat types. (a) The breakdown of eDNA survey sites within each habitat type. Note, that no fish DNA was detected at site KO. (b) Percentage reads merged by habitat type.

Following the application of quality controls, a total of 9,687,953 DNA reads were assigned to sample level (5,949,817 fish reads; 61.4%), averaging 74,522 total reads (45,768 fish reads) per sample. Of the 130 eDNA samples in this study, 31 (23.8%) were positive for *A. anguilla*, presenting relatively high eel reads (Range: 341–154,303). The genus *Oreochromis* was omitted from our dataset due to identifying contamination in several blanks. Following this, across our 35 negative controls (negative, filtration blanks, and extraction blanks), 14 reads of *A. anguilla*, 14 reads of *Rutilus rutilus* and 4 reads of *Carassius* were detected in single filtration blanks. This is indicative of a low level of contamination, and we are therefore confident that our low‐frequency reads threshold eliminates any risk of false positives arising in our dataset. This, therefore, confirms the presence of *A. anguilla* at 11/26 of our study sites (Table 1, Figure 2a). The 11 sites identified as positive for *A. anguilla* included four dam outlets, two reservoirs, two unregulated rivers, two spring‐fed sites and one wetland (Figure 2a). The relative abundance and fish community detected is variable by site and freshwater habitat type (Figure 2). Combined site data show similarities in fish community composition between dam outlets and reservoirs (Figure 2b). Both are dominated by *R. rutilus* and share most other species, notably *A. anguilla* was detected with a higher relative read count and frequency below dams. The overall species community appears reduced by comparison in unregulated rivers, spring‐fed, and wetland sites. Though it should be noted fewer of these habitat sites were sampled. Despite this, *A. anguilla* was detected in all freshwater habitat types (Figure 2a).

### 3.2 Fish monitoring program

**FIGURE 3:**
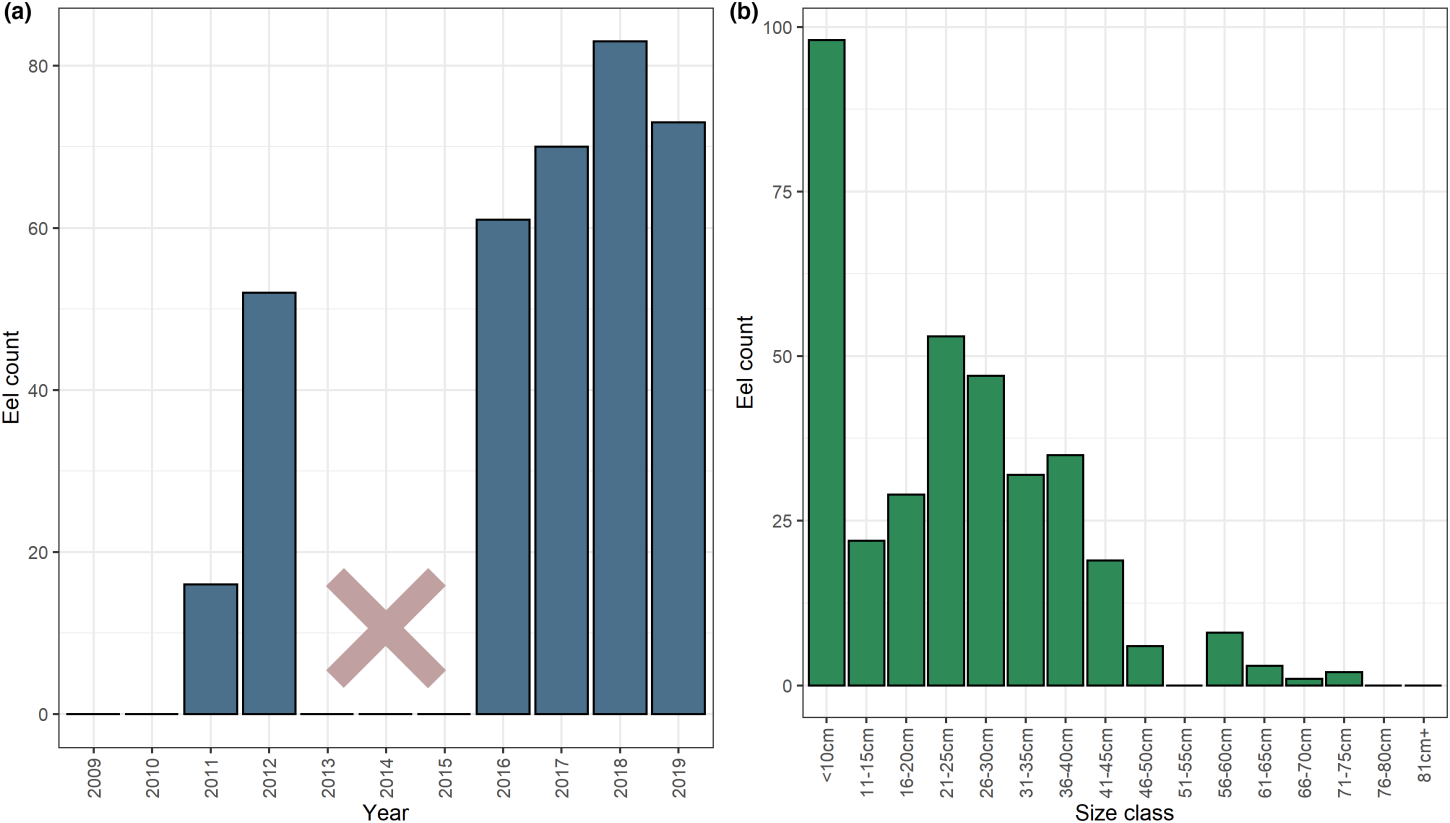
(a) The number of *Anguilla anguilla* individuals captured each survey year, and (b) the size classes of *A. anguilla* captured. Note, that no fish surveys were carried out from 2013–2015, and no eels were captured in 2009–2010 surveys.

The 299 surveys captured a total of 355 *A. anguilla* specimens over the 10‐year period (2009–2019). Of the eels captured during this period, a large proportion of individuals were caught in recent years (Figure 3). Though this may be, at least partly, due to progressive advances in knowledge of fish distribution and stream typology (Dörflinger, 2016), thus leading to more targeted survey planning. Size classes suggest that the majority of eels captured were <50 cm (Figure 3).

**FIGURE 4:**
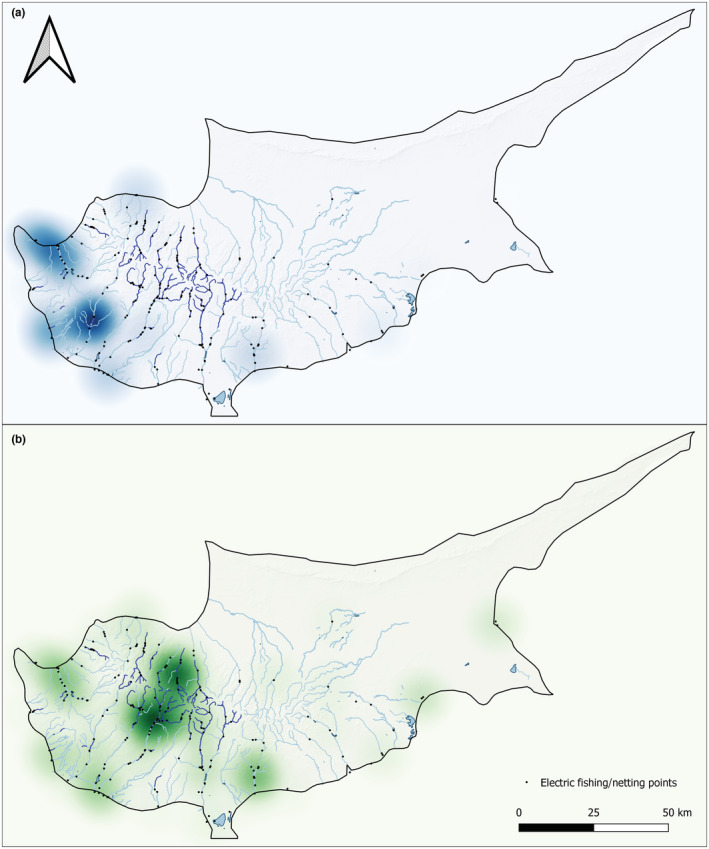
Heatmaps from fish surveys using Kernel Density Estimation, weighted by (a) eel catch and (b) species richness respectively.

Overall, eel presence was confirmed in 61 of the 299 electrofishing/netting surveys (20.4%), mostly distributed in lowland systems on the western side of the island (Figure 4a). It should be noted however, that many of these positive surveys were due to repeat visits of the same catchments. Fish species richness appeared to be concentrated further inland, beyond the current distribution of *A. anguilla* (Figure 4b).

### 3.3 Refuge traps

No glass eels were found in 2020, despite traps being regularly checked beyond our main eDNA sampling period (Table 1). However, in 2019 between 13/03/19 and 04/04/19, a total of 48 glass/pigmented eels were captured ranging from 50 to 66 mm in length, confirming this method was effective when deployed in these catchments. This indicates that water sampling at key eDNA sites in 2020 occurred before eel recruitment that year.

### 3.4 Distribution of eels (combined methods)

**FIGURE 5:**
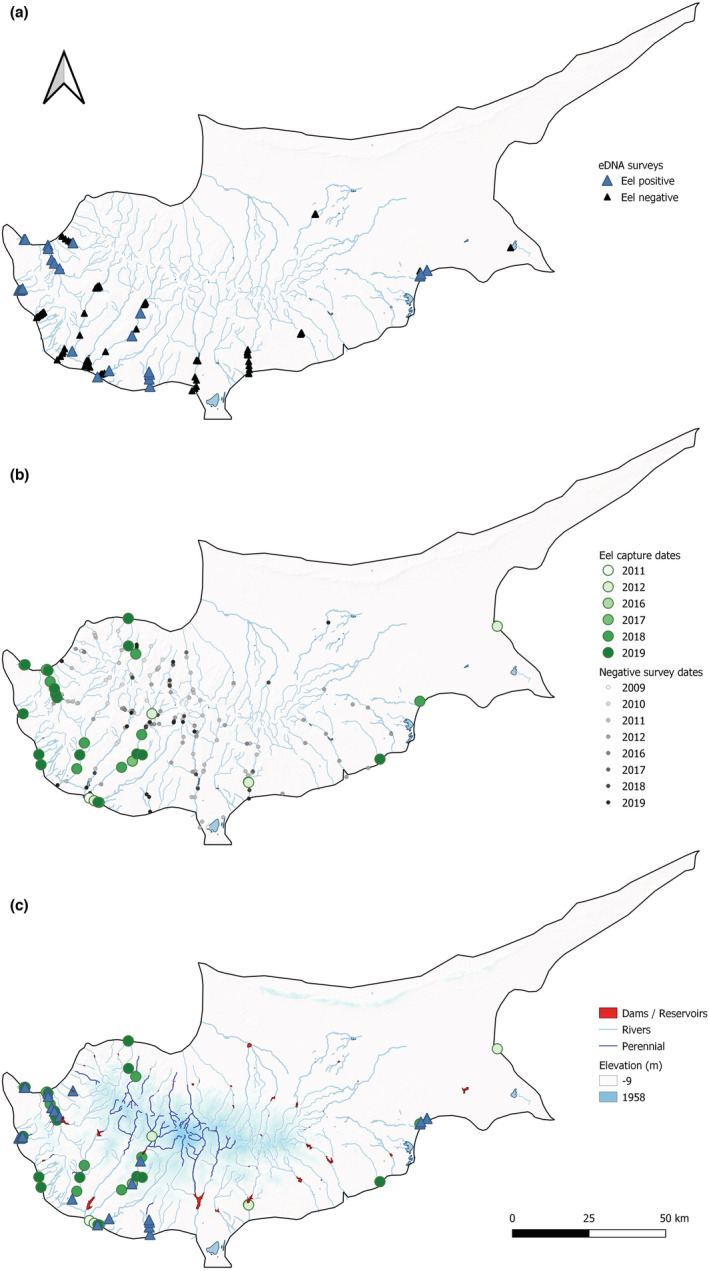
The distribution of *Anguilla anguilla* based on (a) eDNA surveys, (b) electrofishing/netting, and (c) combined positive detections.

**FIGURE 6:**
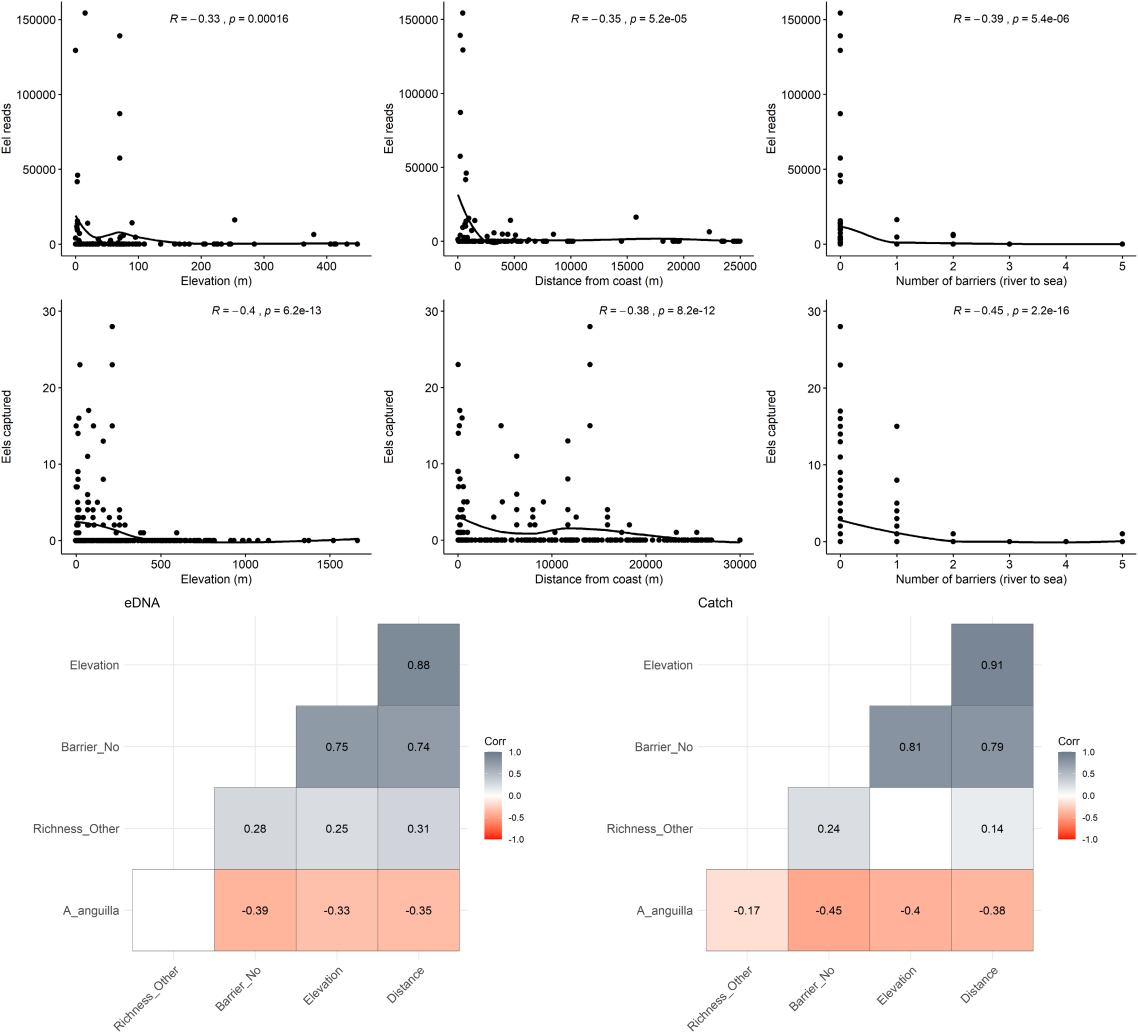
Scatter plots (top) including the Spearman's rank correlation outputs, and smooth curves (loess) to visualise *Anguilla anguilla* associations. Including a correlation matrix to visualise all eDNA based (bottom left) and catch based (bottom right) associations, blank values indicate no significance (*p* = >.05).

**FIGURE 7:**
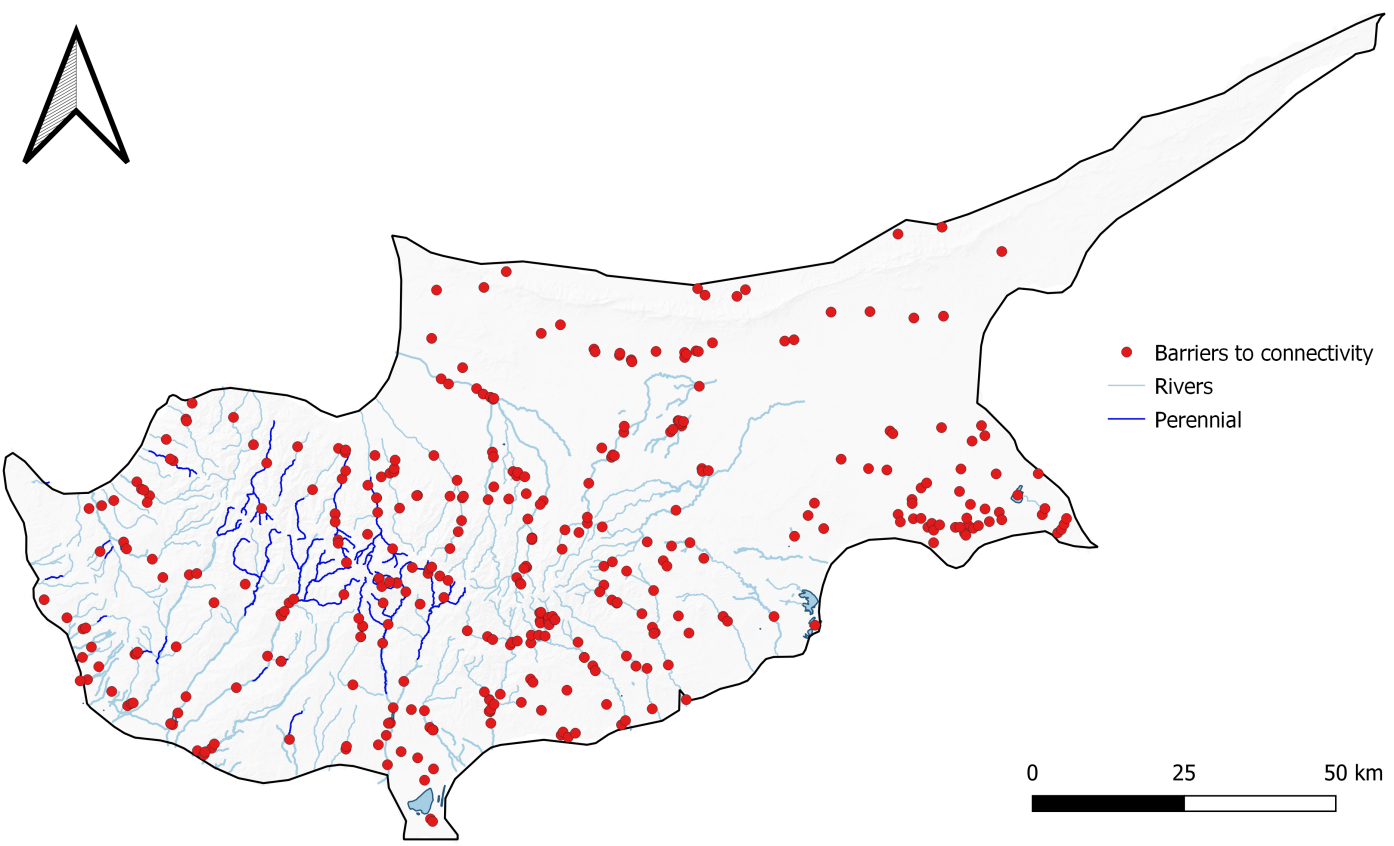
The distribution of artificial instream barriers in Cyprus, based on data from the AMBER Consortium (2020).

Integrated monitoring gives us the best idea of overall eel distribution, and this shows a consistent pattern (Figure 5c). While both methods were able to detect *A. anguilla* up to 25 km inland (Figure 5), water retention dams appear to be acting as barriers, preventing eels from accessing the central regions where overall species richness is highest (Figure 4). In these upper reaches, eel was present historically (Zogaris et al., 2012), but from the 299 fish surveys, only 1 eel was confirmed in this region in 2012 (Figure 5b). Excluding this record, and the 2/9 reservoirs positive for eel eDNA (Figures 2 and 5c), no other eels have been confirmed above dam structures. Both eDNA and catch methods found that eels were negatively correlated with elevation (*R* = −.33, *p* = <.05; *R* = −.4, *p* = <.05), distance from coast (*R* = −.35, *p* = <.05; *R* = −.38, *p* = <.05), and number of barriers (*R* = −.39, *p* = <.05; *R* = −.45, *p* = <.05), respectively (Figure 6). Overall species richness presented weak positive associations with the number of barriers, based on eDNA (*R* = .28, *p* = <.05) and catch (*R* = .24, *p* = <.05) methods (Figure 6). It is noted that the number of barriers, elevation, and distance from coast was all positively associated (Figure 6). Artificial instream barriers are widespread throughout Cyprus (Figure 7) (AMBER Consortium, 2020), yet their impacts have remained largely undocumented. In addition to the 108 dams, culverts (*n* = 34) and weirs (*n* = 42) are widespread on the island. While an additional 176 barriers are classified as “other,” suggesting no operational purpose, or that current information is lacking.

## 4 DISCUSSION

### 4.1 Eels in Cyprus

Our study confirms the presence of *A. anguilla* in Cyprus' inland freshwaters. However, these results highlight that present‐day eel distribution is restricted to lower elevation freshwater systems, primarily distributed in the western part of the island with close proximity to the coast. The freshwaters of Cyprus have largely been overlooked in regard to *A. anguilla* at policy level, and historically quantitative data regarding their distribution, presence and status have been lacking. We build on the initial study by Zogaris et al. (2012), which reported eels were historically widespread in Cyprus based on literature reviews and expert interviews, but only physically captured/observed eels in three river basins. Our work applies integrated monitoring to expand on this, reporting present‐day eel occupancy in a range of habitat types using eDNA monitoring, and widespread eel catches in the western lowlands. There was agreement between eDNA and catch surveys in terms of identifying eel “hot spots,” with two catchments in the west of the island consistently positive for *A. anguilla*. Although, it should be acknowledged that the eDNA surveys did not cover the full spatial extent of the 10‐year monitoring program, and thus these trends are not obtained from direct comparisons. With the knowledge that present‐day eel distribution is restricted, we must consider that several streams historically documented as perennial have been recently observed to be in artificially intermittent states (Zogaris et al., 2012). This would suggest that, in recent years, the combination of barriers and widespread water retention upstream of dams is putting pressures on downstream lotic systems. Such pressures, including artificial intermittence, have led to fish kills in summer months. This was reported in 2013 (Zogaris, 2014) and 2021 (WDD, unpublished data), leading to high levels of eel mortality. This highlights that in the absence of access to perennial refugia, eels detected in lowland systems may be at risk.

Similarly to our study, the majority of eels observed during these fish kill events were under 45 cm in size (Zogaris, 2014). The data we present from electrofishing/netting surveys were collected after the spring influx of glass eels, and therefore may be biased toward same year recruitment. However, with so few eels captured measuring >50 cm, there are indications that large female silver eels of the expected spawning size (~65 cm) remain rare (Clevestam et al., 2011). With that in mind, eDNA sampling was carried out before the elver run had peaked in the region (section 3.3). The eDNA detections from 2020 are therefore likely from eels which arrived in previous years, providing novel insight into the distribution of post‐elver stage eels on the island.

The 2020 eDNA surveys detected eel presence in all habitat types. Including the detection of eels upstream of two reservoir dams (Table 1), this is novel information, since electrofishing is not carried out in reservoirs. Aside from these detections, and the one eel captured in 2012 in a stream above dam structures, no other eels were found upstream of dams. Considered together, this provides evidence that while eels are entering Cyprus' freshwater systems, they are likely restricted by dams and other barriers to lowland intermittent systems, where connectivity to summer refuge is limited and often restricted to relatively short spring‐fed reaches (Dörflinger, 2016). These new insights highlight that despite widespread inland historic records from expert interviews and questionnaires (Zogaris et al., 2012), eels are not currently present in the central and higher elevation regions of the island, likely due to the many barriers to connectivity. While the central regions of the island were host to other fish species, it is apparent that currently, connectivity is not sufficient for eels to access them. Despite DNA reads and individuals caught not being comparable measures of abundance, eDNA and catch data drew parallel conclusions, since both methods found eel presence was negatively correlated to distance from sea, elevation, and number of artificial barriers. It should be noted, however, that given these three variables are strongly correlated, it is difficult to discern their relative impact independently. Eel presence is largely influenced by upstream passability (Teichert et al., 2020), and while eels are able to navigate some barriers to connectivity (Halvorsen et al., 2020), the cumulative impact of many barriers, in addition to large water retention dams, appears to be restricting their distribution. It is therefore not unexpected that more eels are found closer to the tidal limit, given their catadromous lifecycle.

Cyprus is a densely populated and semi‐arid island, meaning that water stress on aquatic biodiversity is often unavoidable. As a result, the 108 water retention dams are relied upon to enable water management, with a capacity of 331,951,000 m^3^ (WDD, 2017). With water scarcity considered an enduring issue, which may be further exacerbated by climate change, such pressures are hard to remove completely. There are, however, many artificial barriers to eel and fish passage, which may be more easily mitigated (AMBER Consortium, 2020). By increasing connectivity within the lower reaches of catchments, the impact of upstream pressures may be diluted as more downstream habitat is available in the event water retention leads to an artificially intermittent environment in systems with previously perennial flows. Recent work by Dörflinger (2016) found the island's river network is composed of 14% perennial and 86% intermittent stretches. Most perennial sites were classified as mountain streams, with an average elevation of 1051 m, although fragmented stretches of perennial freshwaters at lower elevations are present. Until recently the distribution and extent of perennial streams were unknown, and these improvements in knowledge of river typology will aid future planning of targeted monitoring and management actions.

Quantifying the passability and impacts of specific barriers was beyond the scope of this study. In the face of increasing pressure from barriers and anthropogenic impacts in Mediterranean freshwaters, up‐to‐date knowledge on site specifics is hard to feed into a robust management plan. Our data suggest that while barriers have a significant impact on eel distribution, in the absence of sufficient data, eel priority sites could be identified based on elevation and distance from coast. Based on our findings, we suggest that lowland intermittent systems with limited connectivity should be routinely monitored for eels as part of any future eel management plans. With the recommendation, that connectivity is increased between intermittent systems and perennial refuge, in addition to ensuring river to sea connectivity. This would increase the likelihood of eels accessing freshwater refuge, while allowing them to escape systems prone to desiccation and enabling silver eel migration.

### 4.2 Wider implications

Environmental DNA metabarcoding was able to detect our target species *A. anguilla* and fish communities in highly modified Mediterranean freshwater systems. In addition, the 2020 eDNA surveys were able to uncover widespread eel distribution, which broadly reflected the 10‐year electrofishing surveys. This highlights how eDNA metabarcoding can be applied successfully as part of an integrated monitoring programme in such systems. Allowing for a wide range of sites to be surveyed in a short time period, without compromising on detection rates. We suggest that eDNA metabarcoding of water samples would be a valuable tool in the implementation of any future Mediterranean eel management plans. By enabling wide‐scale sampling to identify eel status, and therefore focus limited resources on eel priority catchments. There are however some caveats to consider, mainly concerning the downstream transportation of eDNA. This can be observed in our eDNA data in this study, where due to downstream transportation of eDNA (Deiner et al., 2016; Milhau et al., 2021; Pont et al., 2018) and the potential for fish overspill/migration, reservoirs and their associated downstream outlets were found to have similar species compositions. When considering this for eel management, it is therefore important to interpret such data at a catchment scale. This explains why when plotting species richness spatially, only catch data are included, in order to ensure spatially precise distributions. There are also of course situations when physically handling the target species is required, in order to generate size class data, for example. We, therefore, suggest that eDNA‐based monitoring is applied to systems with limited knowledge of eel distributions, with traditional surveys then targeting sites designated as eel priority. Combining both eDNA sampling and catch methods in an exploratory manner, to cover more terrain rapidly, may work best.

Our results are reflective of wider pressures on freshwater catchments in south and east Europe, where conservation action is required due to growing threats from river regulation, dam construction, hydropower and climate change (Szabolcs et al., 2022). Steps to identify eel priority sites, improve connectivity to water supply dams where possible, and consider eel passability in the design for future dams during the construction phase could be of benefit here. Given the recent GFCM recommendation, GFCM/42/2018/1 (GFCM, 2018) to establish targeted management for *A. anguilla* in the Mediterranean Sea (ICES, 2021), alongside the knowledge that IRES are increasing in number in semi‐arid regions (Datry et al., 2014, 2018), including the emergence of artificially intermittent river systems in the Mediterranean (Skoulikidis et al., 2011). The process of rethinking eel management plans in Cyprus could be applied to inform other semi‐arid regions in the development of future eel management strategies in freshwaters.

## 5 CONCLUSIONS

The findings of this study highlight the presence of *A. anguilla* in Cyprus' freshwater systems, and identify that their present‐day distribution is restricted. We present evidence that a critical problem is artificial river corridor fragmentation, caused by a high number of dams, weirs, culverts and other barriers. This, therefore, presents a case for the promotion of river connectivity restoration. Furthermore, the most stable and productive freshwater habitats are often upstream of dams, and therefore opportunities to improve eel passability here should be considered. Achieving this would in part take steps toward the implementation of an eel management plan, even if not in an official capacity. We, therefore, propose that targeted mitigation measures could be applied based on the data from this work, and should initially prioritize fragmented lowland intermittent systems, with known eel presence. In addition, we propose environmental DNA metabarcoding of water samples as a method to complement wide‐scale fish monitoring programs in the Mediterranean. Due to the scattered and low‐density eel populations in the regions' freshwaters, eDNA methods could provide information on the presence of eels in remote and hard to sample areas. There has already been significant work put into the development of a fish bioindicator framework for Cyprus river basins (Zogaris et al., 2012). Effective monitoring of *A. anguilla* could be a key part of this, acting as an indicator of river‐to‐sea connectivity and well‐connected perennial refuge areas further inland. This information is important at a broader scale, at the easternmost range of *A. anguilla*, where more localized eel management plans are now emerging (ICES, 2021). Environmental DNA‐based assessments of eel status could therefore help inform the status of inland freshwaters, while in addition allowing targeted eel management plans to be developed and implemented in areas sustaining present‐day eel populations.

## AUTHOR CONTRIBUTIONS

**Nathan Paul Griffiths:** Conceptualization (supporting); data curation (equal); formal analysis (lead); funding acquisition (equal); investigation (equal); methodology (lead); project administration (equal); visualization (lead); writing – original draft (lead); writing – review and editing (lead). **Rosalind M. Wright:** Conceptualization (lead); data curation (equal); funding acquisition (equal); project administration (equal); resources (lead); supervision (equal); writing – original draft (equal); writing – review and editing (equal). **Bernd Hänfling:** Conceptualization (equal); funding acquisition (equal); project administration (equal); supervision (equal); writing – original draft (equal); writing – review and editing (equal). **Jon Bolland:** Conceptualization (equal); funding acquisition (equal); project administration (equal); supervision (equal); writing – original draft (equal); writing – review and editing (equal). **Katerina Drakou:** Formal analysis (supporting); investigation (supporting); methodology (supporting); writing – original draft (supporting); writing – review and editing (supporting). **Graham S. Sellers:** Data curation (supporting); formal analysis (supporting); methodology (supporting); resources (supporting); software (lead); writing – original draft (supporting); writing – review and editing (supporting). **Stamatis Zogaris:** Investigation (equal); methodology (equal); resources (equal); writing – original draft (equal); writing – review and editing (equal). **Iakovos Tziortzis:** Data curation (equal); investigation (equal); project administration (equal); resources (equal); writing – original draft (equal); writing – review and editing (equal). **Gerald Dörflinger:** Data curation (equal); investigation (equal); project administration (equal); resources (equal); writing – original draft (equal); writing – review and editing (equal). **Marlen I. Vasquez:** Conceptualization (lead); data curation (equal); funding acquisition (equal); investigation (equal); methodology (equal); project administration (equal); resources (equal); supervision (lead); writing – original draft (equal); writing – review and editing (equal).

## FUNDING INFORMATION

DNAqua‐Net (CA15219), UK Environment Agency, Cyprus University of Technology, University of Hull.

## CONFLICT OF INTEREST STATEMENT

The authors declare no conflicts of interest.

### OPEN RESEARCH BADGES

This article has earned an Open Data badge for making publicly available the digitally‐shareable data necessary to reproduce the reported results. The data is available at https://doi.org/10.5281/zenodo.6617377.

## Supporting information


Appendix S1

## Data Availability

Raw sequence reads have been archived on the NCBI Sequence Reads Archive (SRA) under BioProject ID: PRJNA844405. All scripts and corresponding data have been archived and made available at Zenodo: https://doi.org/10.5281/zenodo.6617377.

## References

[ece39800-bib-0001] Aalto, E. , Capoccioni, F. , Terradez Mas, J. , Schiavina, M. , Leone, C. , De Leo, G. , & Ciccotti, E. (2016). Quantifying 60 years of declining European eel (*Anguilla Anguilla* L., 1758) fishery yields in Mediterranean coastal lagoons. ICES Journal of Marine Science, 73(1), 101–110.

[ece39800-bib-0002] Acou, A. , Laffaille, P. , Legault, A. , & Feunteun, E. (2008). Migration pattern of silver eel (*Anguilla Anguilla*, L.) in an obstructed river system. Ecology of Freshwater Fish, 17(3), 432–442.

[ece39800-bib-0003] AMBER Consortium . (2020). The AMBER Barrier Atlas. A Pan‐European database of artificial instream barriers. Version 1.0 June 29th 2020. https://amber.international/european‐barrier‐atlas/

[ece39800-bib-0004] Bilotta, G. S. , Sibley, P. , Hateley, J. , & Don, A. (2011). The decline of the European eel *Anguilla anguilla*: Quantifying and managing escapement to support conservation. Journal of Fish Biology, 78(1), 23–38. 21235544 10.1111/j.1095-8649.2010.02830.x

[ece39800-bib-0005] Bolland, J. D. , Murphy, L. A. , Stanford, R. J. , Angelopoulos, N. V. , Baker, N. J. , Wright, R. M. , Reeds, J. D. , & Cowx, I. G. (2019). Direct and indirect impacts of pumping station operation on downstream migration of critically endangered European eel. Fisheries Management and Ecology, 26(1), 76–85.

[ece39800-bib-0006] Buysse, D. , Mouton, A. M. , Baeyens, R. , & Coeck, J. (2015). Evaluation of downstream migration mitigation actions for eel at an Archimedes screw pump pumping station. Fisheries Management and Ecology, 22(4), 286–294.

[ece39800-bib-0007] Clevestam, P. D. , Ogonowski, M. , Sjöberg, N. B. , & Wickström, H. (2011). Too short to spawn? Implications of small body size and swimming distance on successful migration and maturation of the European eel *Anguilla anguilla* . Journal of Fish Biology, 78(4), 1073–1089. 21463308 10.1111/j.1095-8649.2011.02920.x

[ece39800-bib-0008] Correia, M. J. , Costa, J. L. , Antunes, C. , De Leo, G. , & Domingos, I. (2018). The decline in recruitment of the European eel: New insights from a 40‐year‐long time‐series in the Minho estuary (Portugal). ICES Journal of Marine Science, 75, 1975–1983. 10.1093/icesjms/fsy073

[ece39800-bib-0009] Council of the European Union . (2007). Council Regulation (EC) No 1100/2007 of 18 September 2007 establishing measures for the recovery of the stock of European eel. Official Journal of the European Union L, 248(22.29).

[ece39800-bib-0013] Dörflinger, G. (2016). A new spatial basis for rivers monitoring and management in Cyprus”, DProf thesis. Middlesex University Available from: http://eprints.mdx.ac.uk/20817/

[ece39800-bib-0010] Datry, T. , Boulton, A. J. , Bonada, N. , Fritz, K. , Leigh, C. , Sauquet, E. , Tockner, K. , Hugueny, B. , & Dahm, C. N. (2018). Flow intermittence and ecosystem services in rivers of the Anthropocene. The Journal of Applied Ecology, 55(1), 353–364. 29681651 10.1111/1365-2664.12941 PMC5907507

[ece39800-bib-0011] Datry, T. , Larned, S. T. , & Tockner, K. (2014). Intermittent rivers: A challenge for freshwater ecology. Bioscience, 64(3), 229–235.

[ece39800-bib-0048] Deiner, K. , Fronhofer, E. A. , Mächler, E. , Walser, J.‐C. , & Altermatt, F. (2016). Environmental DNA reveals that rivers are conveyer belts of biodiversity information. Nature Communications, 7, 12544. 10.1038/ncomms12544 PMC5013555 27572523

[ece39800-bib-0012] Dekker, W. (2000). The fractal geometry of the European eel stock. ICES Journal of Marine Science, 57(1), 109–121.

[ece39800-bib-0014] General Fisheries Commission for the Mediterranean (GFCM) . (2018). Recommendation GFCM/42/2018/1 on a multiannual management plan for European eel in the Mediterranean Sea. Issued by the general fisheries Commission for the Mediterranean. Available from: http://www.fao.org/gfcm/decisions/en/

[ece39800-bib-0015] Griffiths, N. P. , Bolland, J. D. , Wright, R. M. , Murphy, L. A. , Donnelly, R. K. , Watson, H. V. , & Hänfling, B. (2020). Environmental DNA metabarcoding provides enhanced detection of the European eel *Anguilla anguilla* and fish community structure in pumped river catchments. Journal of Fish Biology, 1, 177–1384. 10.1111/jfb.14497 33460093

[ece39800-bib-0018] Hänfling, B. , Lawson Handley, L. , Read, D. S. , Hahn, C. , Li, J. , Nichols, P. , Blackman, R. C. , Oliver, A. , & Winfield, I. J. (2016). Environmental DNA metabarcoding of lake fish communities reflects long‐term data from established survey methods. Molecular Ecology, 25(13), 3101–3119. 27095076 10.1111/mec.13660

[ece39800-bib-0016] Halvorsen, S. , Korslund, L. , Gustavsen, P. Ø. , & Slettan, A. (2020). Environmental DNA analysis indicates that migration barriers are decreasing the occurrence of European eel (*Anguilla anguilla*) in distance from the sea. Global Ecology and Conservation, 24, e01245.

[ece39800-bib-0017] Handley, L. L. , Read, D. S. , Winfield, I. J. , Kimbell, H. , Johnson, H. , Li, J. , Hahn, C. , Blackman, R. , Wilcox, R. , Donnelly, R. , Szitenberg, A. , & Hänfling, B. (2019). Temporal and spatial variation in distribution of fish environmental DNA in England's largest lake. Environmental DNA, 1(1), 26–39. 10.1002/edn3.5

[ece39800-bib-0019] ICES . (2019). Joint EIFAAC/ICES/GFCM working group on eels (WGEEL). ICES Scientific Reports, 1(50), 177.

[ece39800-bib-0020] ICES . (2021). European eel (*Anguilla anguilla*) throughout its natural range. Report of the ICES Advisory Committee, 2021, 1–16. 10.17895/ices.advice.7752

[ece39800-bib-0021] Jacoby, D. , & Gollock, M. (2014). *Anguilla anguilla. The IUCN Red List of Threatened Species* 2014*: e. T60344A45833138* .

[ece39800-bib-0022] Kassambara, A. (2020). Ggpubr: “ggplot2” Based Publication Ready Plots. R package version 0.2.5 .

[ece39800-bib-0023] Kelly, R. P. , Port, J. A. , Yamahara, K. M. , & Crowder, L. B. (2014). Using environmental DNA to census marine fishes in a large mesocosm. PLoS One, 9(1), e86175. 24454960 10.1371/journal.pone.0086175 PMC3893283

[ece39800-bib-0024] Kitson, J. J. N. , Hahn, C. , Sands, R. J. , Straw, N. A. , Evans, D. M. , & Lunt, D. H. (2019). Detecting host–parasitoid interactions in an invasive lepidopteran using nested tagging DNA metabarcoding. Molecular Ecology, 28(2), 471–483. 29485216 10.1111/mec.14518

[ece39800-bib-0025] Larned, S. T. , Datry, T. , Arscott, D. B. , & Tockner, K. (2010). Emerging concepts in temporary‐river ecology. Freshwater Biology, 55(4), 717–738.

[ece39800-bib-0026] Markogianni, V. , Tzirkalli, E. , Gücel, S. , Dimitriou, E. , & Zogaris, S. (2014). Remote sensing application for identifying wetland sites on Cyprus: Problems and prospects. *Second International Conference on Remote Sensing and Geoinformation of the Environment* (*RSCy2014*), *9229*, 92291U.

[ece39800-bib-0027] McDonald, J. H. (2014). Handbook of biological statistics (3rd ed.). sparky house publishing Available from: http://www.biostathandbook.com/transformation.html

[ece39800-bib-0047] Milhau, T. , Valentini, A. , Poulet, N. , Roset, N. , Jean, P. , Gaboriaud, C. , & Dejean, T. (2021). Seasonal dynamics of riverine fish communities using eDNA. Journal of Fish Biology, 98, 387–398. 31674010 10.1111/jfb.14190

[ece39800-bib-0028] Moriarty, C. , & Dekker, W. (1997). Management of the European eel. Marine Institute. http://oar.marine.ie/handle/10793/197

[ece39800-bib-0029] Økland, F. , Havn, T. B. , Thorstad, E. B. , Heermann, L. , Saether, S. A. , Tambets, M. , Teichert, M. A. K. , & Borcherding, J. (2019). Mortality of downstream migrating European eel at power stations can be low when turbine mortality is eliminated by protection measures and safe bypass routes are available. International Review of Hydrobiology, 104(3‐4), 68–79.

[ece39800-bib-0030] Papastergiadou, E. , Stefanidis, K. , Dorflinger, G. , Giannouris, E. , Kostara, K. , & Manolaki, P. (2016). Exploring biodiversity in riparian corridors of a Mediterranean Island: Plant communities and environmental parameters in Cyprus rivers. Plant Biosystems ‐ An International Journal Dealing with All Aspects of Plant Biology, 150(1), 91–103.

[ece39800-bib-0031] Podgorniak, T. , Blanchet, S. , De Oliveira, E. , Daverat, F. , & Pierron, F. (2016). To boldly climb: Behavioural and cognitive differences in migrating European glass eels. Royal Society Open Science, 3(1), 150665. 26909192 10.1098/rsos.150665 PMC4736947

[ece39800-bib-0046] Pont, D. , Rocle, M. , Valentini, A. , Civade, R. , Jean, P. , Maire, A. , Roset, N. , Schabuss, M. , Zornig, H. , & Dejean, T. (2018). Environmental DNA reveals quantitative patterns of fish biodiversity in large rivers despite its downstream transportation. Scientific Reports, 8(1), 10361. 29991759 10.1038/s41598-018-28424-8 PMC6039509

[ece39800-bib-0032] QGIS Development Team . (2022). QGIS geographic information system. QGIS Association. https://www.qgis.org

[ece39800-bib-0033] Quail, M. A. , Swerdlow, H. , & Turner, D. J. (2009). Improved protocols for the illumina genome analyzer sequencing system. Current Protocols in Human Genetics, 62(1). 10.1002/0471142905.hg1802s62. Chapter 18:Unit 18.2. PMC3849550 19582764

[ece39800-bib-0034] R Core Team . (2019). R: A language and environment for statistical computing. R Foundation for Statistical Computing.

[ece39800-bib-0035] Riaz, T. , Shehzad, W. , Viari, A. , Pompanon, F. , Taberlet, P. , & Coissac, E. (2011). ecoPrimers: Inference of new DNA barcode markers from whole genome sequence analysis. Nucleic Acids Research, 39(21), e145. 21930509 10.1093/nar/gkr732 PMC3241669

[ece39800-bib-0036] Schmidt, J. (1923). The Breeding Places of the Eel. Philosophical Transactions of the Royal Society of London. Series B, Containing Papers of a Biological Character, 211, 179–208.

[ece39800-bib-0037] Sellers, G. S. , Di Muri, C. , Gómez, A. , & Hänfling, B. (2018). Mu‐DNA: A modular universal DNA extraction method adaptable for a wide range of sample types. Metabarcoding and Metagenomics, 2, e24556.

[ece39800-bib-0038] Skoulikidis, N. T. , Vardakas, L. , Karaouzas, I. , Economou, A. N. , Dimitriou, E. , & Zogaris, S. (2011). Assessing water stress in Mediterranean lotic systems: Insights from an artificially intermittent river in Greece. Aquatic Sciences, 73(4), 581–597.

[ece39800-bib-0039] Szabolcs, M. , Kapusi, F. , Carrizo, S. , Markovic, D. , Freyhof, J. , Cid, N. , Cardoso, A. C. , Scholz, M. , Kasperidus, H. D. , Darwall, W. R. T. , & Lengyel, S. (2022). Spatial priorities for freshwater biodiversity conservation in light of catchment protection and connectivity in Europe. PLoS One, 17(5), e0267801. 35580083 10.1371/journal.pone.0267801 PMC9113586

[ece39800-bib-0040] Teichert, N. , Tétard, S. , Trancart, T. , de Oliveira, E. , Acou, A. , Carpentier, A. , Bourillon, B. , & Feunteun, E. (2020). Towards transferability in fish migration models: A generic operational tool for predicting silver eel migration in rivers. The Science of the Total Environment, 739, 140069. 32544695 10.1016/j.scitotenv.2020.140069

[ece39800-bib-0041] Trancart, T. , Carpentier, A. , Acou, A. , Charrier, F. , Mazel, V. , Danet, V. , & Feunteun, E. (2020). When “safe” dams kill: Analyzing combination of impacts of overflow dams on the migration of silver eels. Ecological Engineering, 145, 105741. 10.1016/j.ecoleng.2020.105741

[ece39800-bib-0042] WDD . (2017). Water Development Department ‐ Catalogue of reservoirs . http://www.moa.gov.cy/moa/wdd/wdd.nsf/All/25041A3BAC14BC48C22583D800396FF5?OpenDocument

[ece39800-bib-0043] Wickham, H. (2016). ggplot2: Elegant graphics for data analysis. Springer‐Verlag.

[ece39800-bib-0044] Zogaris, S. (2014). Ichthyological study for Oroklini Lake. April 2014. BirdLife Cyprus. A study within the project LIFE10 NAT/CY/716 ‘restoration and Management of Oroklini Lake SPA, Larnaca’.Unpublished final report, 58 pp. Nicosia, Cyprus.

[ece39800-bib-0045] Zogaris, S. , Chatzinikolaou, Y. , Koutsikos, N. , Economou, A. N. , Oikonomou, E. , Michaelides, G. , Hadjisterikotis, E. , Beaumont, W. R. C. , & Ferreira, M. T. (2012). Freshwater fish assemblages in Cyprus with emphasis on the effects of dams. Acta Ichthyologica et Piscatoria, 42(3), 165–175. https://www.researchgate.net/profile/Maria_Ferreira29/publication/232712643_Freshwater_Fish_Assemblages_in_Cyprus_with_Emphasis_on_the_Effects_of_Dams/links/0046352026b39baa6f000000/Freshwater‐Fish‐Assemblages‐in‐Cyprus‐with‐Emphasis‐on‐the‐Effects‐of‐Dams.pdf

